# Pathogenesis of Children’s Allergic Diseases: Refocusing the Role of the Gut Microbiota

**DOI:** 10.3389/fphys.2021.749544

**Published:** 2021-10-14

**Authors:** Tingting Hu, Yinmiao Dong, Chenghao Yang, Mingyi Zhao, Qingnan He

**Affiliations:** Department of Pediatrics, The Third Xiangya Hospital, Central South University, Changsha, China

**Keywords:** children’s allergic diseases, gastrointestinal (GI) microbiota, asthma, allergic rhinitis, food allergy (FA)

## Abstract

Allergic diseases comprise a genetically heterogeneous cluster of immunologically mediated diseases, including asthma, food allergy (FA), allergic rhinitis (AR) and eczema, that have become major worldwide health problems. Over the past few decades, the spread of allergic diseases has displayed an increasing trend, and it has been reported that 22% of 1.39 billion people in 30 countries have a type of allergic disease. Undoubtedly, allergic diseases, which can be chronic, with significant morbidity, mortality and dynamic progression, impose major economic burdens on society and families; thus, exploring the cause of allergic diseases and reducing their prevalence is a top priority. Recently, it has been reported that the gastrointestinal (GI) microbiota can provide vital signals for the development, function, and regulation of the immune system, and the above-mentioned contributions make the GI microbiota a key player in allergic diseases. Notably, the GI microbiota is highly influenced by the mode of delivery, infant diet, environment, antibiotic use and so on. Specifically, changes in the environment can result in the dysbiosis of the GI microbiota. The proper function of the GI microbiota depends on a stable cellular composition which in the case of the human microbiota consists mainly of bacteria. Large shifts in the ratio between these phyla or the expansion of new bacterial groups lead to a disease-promoting imbalance, which is often referred to as dysbiosis. And the dysbiosis can lead to alterations of the composition of the microbiota and subsequent changes in metabolism. Further, the GI microbiota can affect the physiological characteristics of the human host and modulate the immune response of the host. The objectives of this review are to evaluate the development of the GI microbiota, the main drivers of the colonization of the GI tract, and the potential role of the GI microbiota in allergic diseases and provide a theoretical basis as well as molecular strategies for clinical practice.

## Introduction

The World Health Organization (WHO) has identified allergic diseases as one of the three major diseases that need focal prevention and a cure; moreover, allergic diseases are considered a current serious global problem (Nwaru and Virtanen, 2017). Allergic diseases, mainly asthma, food allergy (FA), allergic rhinitis (AR), eczema and so on, are traditionally referred to as immediate or type 1 hypersensitivity reactions, with IgE as a critical factor (Han et al., 2020; Justiz Vaillant et al., 2021). This kind of anaphylactic reaction is mediated by IgE antibodies that are produced by the immune system in response to environmental proteins which are termed allergens including pollens, animal danders, or dust mites. These IgE antibodies bind to mast cells and basophils, which include histamine particles released during the reaction, and eventually cause inflammation (Crosson et al., 2021; Justiz Vaillant et al., 2021; Shamji et al., 2021). According to the World Allergy Organization (WAO)^1^, an epidemiological survey on allergic diseases showed that 22% of the population in 30 countries has a type of allergic disease. A study in 2003 indicated that the morbidity of asthma among children under 4 years old in the United States has increased by 160% since the 1980s and 1990s (Eichenfield et al., 2003). According to a cross-sectional study of the whole population in six regions of Inner Mongolia, northern China, the report said that 4,441 of the respondents (18.0%) self-reported food allergies, and the incidence rate of children was higher than that of adults (38.7 vs. 11.9%, *P* = 0.335, respectively) (Wang et al., 2018). The incidence of major allergic diseases, i.e., asthma, allergic rhinitis and eczema, in the urban centers of Peking, Chongqing, and Guangzhou are summarized in Table 1 (Zhao et al., 2011). As shown, the incidence rate of asthma in Peking, Chongqing, and Guangzhou were 3.15, 7.45, and 2.09%, respectively; the incidence of allergic rhinitis was 14.46, 20.42, and 7.22%, respectively; and the self-reported in of eczema was 20.64, 10.02, and 7.22%, respectively (Zhao et al., 2011). According to a cross-sectional study aimed at children aged 0–14 years in Hong Kong, China, 352 (4.8%; 95% CI 4.3–5.3%) of 7,393 children were reported to suffer from FA (Ho et al., 2012). In addition, the above-mentioned tables all show that the morbidity of allergic diseases was higher in males than in females. In general, the incidence rate of allergic diseases continues to rise, particularly in Asia (Hong et al., 2004; Anandan et al., 2010; Wong et al., 2013). Therefore, exploring the cause of allergic diseases and reducing the incidence of allergic diseases are of vital importance.

**TABLE 1:** Incidence of allergic diseases in children of different cities and genders (Zhao et al., 2011).

	Asthma			Allergic rhinitis			Eczema		
	Overall	Male	Female	Overall	Male	Female	Overall	Male	Female
Peking	3.15%	4.09%	2.05%	14.46%	16.44%	12.26%	20.64%	21.13%	20.09%
Chongqing	7.45%	8.22%	6.06%	20.42%	22.08%	18.53%	10.02%	11.34%	10.66%
Guangzhou	2.55%	3.87%	1.60%	7.22%	8.93%	6.55%	7.22%	7.52%	6.87%

*Data are n (%) unless otherwise stated. The data are from reference (Zhao et al., 2011).*

**FIGURE 1:**
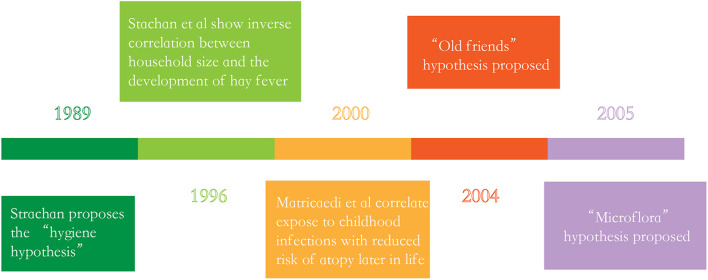
The history of the “hygiene hypothesis”.

The “hygiene hypothesis” proposed by Strachan in the late 1980s is currently considered the main theory targeted to explain the peculiar generational rise in immune dysregulation (Strachan, 1989), this hypothesis argues that a lack of exposure to infectious sources, parasites and symbiotic microorganisms such as the GI microbiota limits the normal development of the immune system, ultimately resulting in the increasing incidence of allergic diseases (Hong et al., 2004; Anandan et al., 2010; Wong et al., 2013). However, research over the past decade has provided evidence which linked the commensal and symbiotic microbes (GI microbiota) and parasitic worms to immune development, extending the hygiene hypothesis to the “microflora” and “old friends” hypotheses, respectively (Figure 1; Rook et al., 2004; Noverr and Huffnagle, 2005). Rook et al. (2004) proposed the “old friends” hypotheses. This hypotheses argues that microorganisms and macroorganisms such as parasitic helminths co-evolved with the development of the human immune system, and it also notes that these organisms are necessary for normal immune system development, which are similar to the “hygiene hypothesis” (Rook et al., 2004; Stiemsma et al., 2015). In 2005, the “microflora” hypothesis is proposed. In fact, the “microflora” hypothesis is considered as an another modern extension of the “hygiene hypothesis,” which indicates that early life disturbance (by antibiotic use, infection, or diet) to the bacteria which resides in the human intestine (the GI microbiota) destroy the normal microbial mediated mechanisms promoting immunological tolerance, and finally makes the immune system toward a state of promoting allergic disease (Noverr and Huffnagle, 2005; Bae, 2018).

Now, we have a much clearer understanding of the interactions between the GI microbiota and immune system with the emergence of next-generation sequencing (NGS) and personal genome sequencing. This review aims to summarize the research progress on the relationship between the GI microbiota and children’s allergic diseases, such as asthma, allergic rhinitis, eczema, and FA; in addition, it provides novel thoughts for preventive strategies and the treatment of allergic diseases.

## The Development of the Gut Microbiota

### The Composition of the Gut Microbiota

In healthy individuals, there is a diverse microbial community present in the gut with numerous bacterial species, defined as the microbiota (Harmsen and de Goffau, 2016). Research has shown that the human gut microbiota likely contains 1,000–1,500 bacterial species; nevertheless, each person has only approximately 160 bacterial species (Lee and Mazmanian, 2010; Shi et al., 2017). Tierney et al. (2019) made an effort to sequence the bacterial genomes of the microbiota, indicating that our gut microbiota contains about 22.2 million genes, which is over 700 times the length of the human genome. The GI microbiota of healthy individuals was primarily composed of four phyla of bacteria: *Firmicutes, Bacteroidetes, Proteobacteria*, and *Actinobacteria* (Wright et al., 2015). Furthermore, most of the microbiota was composed of the phyla *Firmicutes* and *Bacteroidetes*, and the class *Clostridia* dominated the *Firmicutes* sequences (Cai et al., 2021). Of note, the microbiome includes not only bacteria, but also viruses, phage, fungi, archaea and so on (Gong et al., 2021).

### Origins of Fetal Microbiota and Maternal Gut Microbiota

In the past decades, numerous studies on the GI microbiota have been restricted to the point of view that fetuses, considered microbiologically sterile, would not be exposed to bacteria until they come into contact with the vaginal and intestinal microbiota of the mother or with other environmental microorganisms at birth (Satokari et al., 2009). Nevertheless, this traditional view has been challenged by a new view that microbial colonization of the healthy new-born intestinal tract may begin *in utero* rather than during or after birth (Milani et al., 2017). Studies have found that the placenta harbors a unique microbiota composed of non-pathogenic commensal microbiota, which is similar to the oral microbiota of the mother, indicating that the placental microbiota may be established by the hematogenous spread of oral microbiota (Aagaard, 2014; Prince et al., 2016; Theis et al., 2019). Jimenez et al. (2008) detected microbial DNA in meconium (Mshvildadze et al., 2010). An additional study has found a high degree of similarity between microbes in meconium and those in amniotic fluid; presumably, this is due to the high likelihood that fetuses swallow plentiful amounts of amniotic fluid during the later stages of pregnancy, and meconium microbes are derived from the amniotic fluid that is swallowed (Nanthakumar et al., 2000; Ardissone et al., 2014; Cereta et al., 2021). Taken together, these finding show that microbial colonization of the healthy new-born GI tract may begin *in utero*.

While there is good evidence that prenatal exposure to maternal gut microbes may occur, the mechanisms by which microbes may be transported from the maternal GI to the developing fetus have yet to be identified. At present, the most likely hypothesis is that microbes are transferred from the intestinal epithelium to the bloodstream and then transported to the placenta. Normally, an epithelial barrier prevents bacteria from entering the bloodstream; however, dendritic cells (DCs) have been shown to actively penetrate intestinal epithelial cells and absorb bacteria from the intestinal lumen. Studies have shown that DCs can isolate living symbiotic bacteria here for a few days. Once attached to dendritic cells and transferred to the lymphatic system, the bacteria can spread to other parts of the body (Laubereau et al., 2004). These bacteria-loaded dendritic cells can be transported to mesenteric lymph nodes through GI lymphatic vessels (Jimenez et al., 2008). GI microbiota are also found in breast milk, and the same transport mechanism has been theorized (Forsberg et al., 2013).

However, it is of importance to be mindful that the above-mentioned source may not be the only one, that may vary between pregnancies and that fetal seeding may be a dynamic, gestational age-dependent process. And by determining the origins of the fetal microbiome, we might find chances for pre- and post-natal health interventions to prevent dysbiosis and minimize the incidence and impact of non-communicable diseases.

Neonates demonstrate a complex microbiota in the gut within the week immediately following birth, with dynamic fluctuations in the composition of the gut microbiota until a stable adult microbiota composition is reached at the age of 2–2.5 years. Research suggests that facultative and aerotolerant bacteria such as lactic acid bacteria, *Enterobacteria*, and streptococci dominate the initial flora in the neonatal gut, followed by an increasing number of strict anaerobes, including *Bifidobacterium, Clostridium, Bacteroides* (Bottacini et al., 2017; Milani et al., 2017; Brosseau et al., 2021).

## Main Drivers of Colonization and Affecting Factors of the Infant Gastrointestinal Tract

**FIGURE 2:**
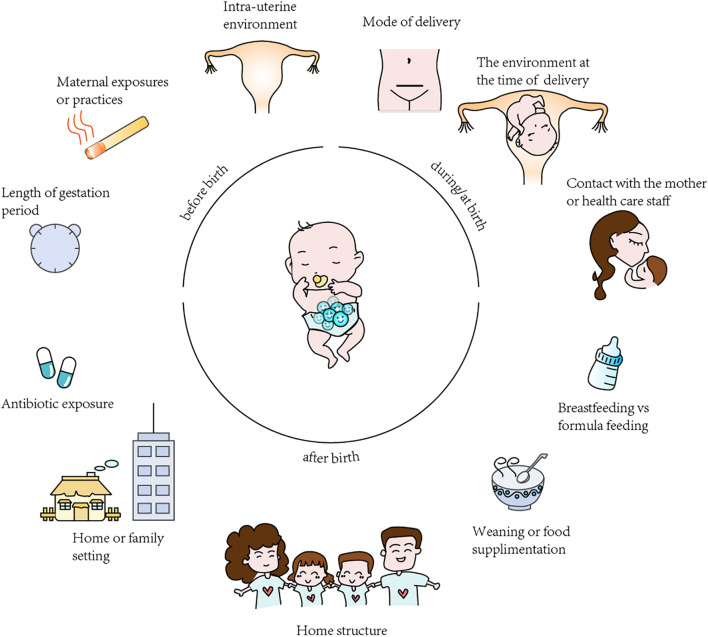
Summary of the factors affecting gut microbiota colonization in infants. The “before birth”, “during birth”, “after birth” factors affecting analysis and results in microbiota studies.

The most important affecting factors of the GI tract composition in infants are divided into those occurring before birth, during/at birth and after birth, with the main factors being the mode of delivery, infant diet (i.e., the type of infant feeding) environment, antibiotic use by the infant and so forth (Figure 2; Azad et al., 2016; Cryan et al., 2019).

### Mode of Delivery

The delivery mode is thought to be a vital driver of the early GI tract composition in full-term infants (Dominguez-Bello et al., 2010). A large number of studies have reported differences in full-term infants gut microbiota diversity and/or composition between caesarean delivery (CD) and vaginal delivery (VD) delivered infants. And consistent among many of these researches are findings of lower overall microbial diversity and decreased abundance of *Bifidobacterium*, *Bacteroides*, and *Lactobacillus* in infants born by CD compared to those VD (Dominguez-Bello et al., 2010; Jakobsson et al., 2014; Brumbaugh et al., 2016; Francino, 2018). For example, Lee et al. (2016) collected fecal samples and performed 16S rRNA gene analysis via 454 pyrosequencing of the V1–V3 regions. Unsurprisingly, they found that during the neonatal period, infants born by caesarean delivery (CD) showed a lower richness and diversity of the GI microbiota than those born by vaginal delivery (VD; Lee et al., 2016).

Differences in microbial colonization patterns are of importance, not only because of their potential impact on the eventual composition of the microbiota, but also because they affect the concomitant development of the infant’s immune system. The maturation of intestinal mucosa and its associated lymphoid tissue depends on intestinal colonization. The GI-associated lymphoid tissue Peyer’s patches, mesenteric lymph nodes, and isolated lymphoid follicles require signals from the GI microbiota to fully develop and/or recruit mature immune cell complement (Maynard et al., 2012). In addition, microbiota shape the immune phenotype in early infancy by binding to molecular receptors in various intestinal immune cells and initiating cascades of signals that indicate cell differentiation and control inflammatory states (Romagnani, 2006). In conclusion, under the influence of the GI microbiota, the immune cell pool established during this critical period will influence the host’s lifetime immunity and susceptibility to disease (Maynard et al., 2012; Francino, 2014; Brugman et al., 2015). Thus, the disruption of colonization caused by CD may be reflected in altered immune system development with potential long-term consequences. Several studies have addressed the impact of CD on T-cell response development and production of different cytokines. The study by Jakobsson et al. (2014) identified limited evidence that CD is thought to be linked with lowered cyclic dynamic levels of Th1-associated chemokines in infancy (Jakobsson et al., 2014). And the study by Jakobsson et al. (2014) showed that *Bacteroides* can exert strong effects on the immune system. In the case of *Bacteroides fragilis*, a surface polysaccharide is recognized by toll-like receptor 2 (TLR2) in Treg cells and induces production of IL-10 and other cytokines that affect the Th1/Th2 balance and promote immune tolerance (Round and Mazmanian, 2010). So the absence of *Bacteroides* may be related to the reduction of Th1 responses detected in CD infants. And poor immune system can result in remarkably lower levels of Th1-associated chemokines, such as CXL10 and CXL11, which are associated with immune responses (Munyaka et al., 2014). Here, it is conjectured that birth via CD is related to adverse effects on immune development and allergies.

### Infant Diet

The type of infant feeding is another profound element affecting the composition and function of the GI microbiota (Cerdo et al., 2019). Human milk oligosaccharides (HMOs) are important components of breast milk and can regulate the proliferation and maturity of GI cells and benefit the growth and development of babies. In addition, HMOs tend to provide nutrients, probiotics, and IgAs, which protect infants from exogenous infections and makes the immune system work more efficiently. Research on the transcription of enterocytes by Praveen et al. (2015) suggests that the infant diet also has a great influence on the expression of host genes and that breastfeeding can lead to the promotion of gene transcription related to immunity and metabolism (Praveen et al., 2015). Furthermore, it is well documented that the GI microbiota of formula-fed infants shows higher diversity than that of breastfed babies (Bezirtzoglou et al., 2011). The GI microbiota of breastfed infants tends to be dominated by *Bifidobacteria* during the first week of life, with members of the *Enterobacteriaceae* family concomitantly decreasing (Cerdo et al., 2019). Therefore, for the first 6 months of life, babies can benefit from exclusively being fed breast milk.

### Antibiotic Use

Antibiotics are the most frequently used drugs in pediatric therapy, nonetheless, excess antimicrobial use and long-term use of antibiotics can not only clear pathogenic microorganisms from the host but can also kill beneficial gut bacteria, induce the alteration of intestinal flora and affect gene expression in the intestine via immunoreactions (Chai et al., 2012; Cox et al., 2014; McArdle et al., 2021). Gasparrini et al. (2019) used 16S rDNA community analysis to study the GI microbiota of 437 infant stools that had taken antibiotics and concluded that these newborns, including 41 preterm infants, had a greater abundance of ***Enterobacter***, ***Escherichia***, and ***Enterococcus***, and a lower relative abundance of ***Bifidobacterium*** during the course of antibiotic use. Furthermore, animal models have demonstrated that antibiotic use can destroy the GI microbiota and subsequently slow the growth of the immune system, which results in airway hyper-responsiveness in susceptible children and thereby increases the risk of allergic diseases such as asthma and allergic rhinitis (Korpela et al., 2016). As mentioned above, even short-term use, especially during the first 2 years of childhood, may lead to long-term changes in the GI microbiota that alter host interactions (Vangay et al., 2015). Gut dysbiosis promotes the horizontal transfer of resistance genes and fuels the evolution of drug-resistant pathogens and the spread of antibiotic resistance (Stecher et al., 2013).

### Environment

Environment, which mostly includes family lifestyle, family members, close siblings, family size and structure, birth order and geographical location, has also been considered a correlative factor that may affect the infant GI microbiota. Some recent studies defined the “sibling effect,” namely, that the anaerobe-to-facultative anaerobe ratio and the abundance of *Bifidobacteria*, in infants with older siblings are higher than those in infants without siblings (Rodriguez et al., 2015). An analysis comparing American children in an upper-middle-class community and Bangladeshi children living in an urban slum of the same age living in poorer conditions indicated that the American children had a greater abundance of *Bacteroides* and were depleted in *Prevotella* than the Bangladeshi children (Lin et al., 2013). These results suggest that differing environmental or genetic factors may shape the microbiota of healthy children in the two countries. Another analysis comparing children from southeastern Africa and northern Europe showed differences in microbiota composition and structure, with the microbiota of African children being enriched in *Bifidobacterium*, *Bacteroides*, and *Prevotella* compared to the microbiota of European children (Lin et al., 2013).

## The Gastrointestinal Microbiota and Allergic Diseases

### Allergic Asthma

Allergic asthma is a complicated chronic respiratory disease (CRD) characterized by airway hyper-responsiveness (AHR) and airway remodeling. Allergic asthma is a global health issue that has affected over 300 million patients of all age groups and all regions worldwide; over the last few decades, the morbidity of allergic asthma has been increasing (Bousquet et al., 2010; McKenzie et al., 2017). Currently, asthma is commonly treated with inhaled corticosteroids (ICSs) and beta-2 adrenergic receptor agonist (i.e., Salbutamol), which can help control symptoms; unfortunately, this treatment is not a cure. Therefore, there is priority in exploring novel, effective therapeutic methods to combat allergic asthma, and the microbiota and its associated metabolites have been considered as a therapeutic target.

**FIGURE 3:**
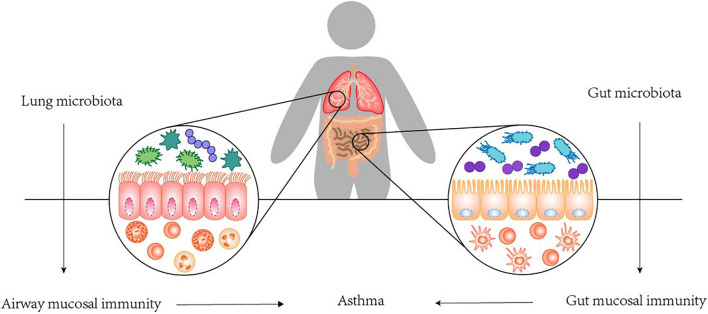
The gut-lung axis.

In fact, the colonization of microorganisms in human lung is closely related to its anatomical and physiological functions. Respiratory microbes that enter the mouth travel to the lungs and are suspended in the air or on particles. The upper respiratory tract is layered with cylindrical respiratory epithelium covered by mucous membrane. The constant fluctuation of mucus and airflow determines the balance between microbial migration and elimination. Mucociliary micromotility and coughing support microbial clearance, all of which are influenced by host immune status (Dickson et al., 2014). It has been proposed that the main source of lower respiratory colonization is the resident upper respiratory microbiome. It also seems plausible that bacteria may reach the lower respiratory tract through micro-inhalation of oropharyngeal secretions and, to a lesser extent, direct inhalation (Segal et al., 2013). And in healthy individuals, low density and continuous renewal of the lung microbiome and low bacterial replication rates have been observed. Conditions conducive to the replication and persistence of certain bacterial species may lead to imbalances or disorders in the lung microbiome, which may lead to the development of asthma (Teo et al., 2015). Colonization of the upper respiratory tract began very early, as tracheal aspirations of newborns only a few hours after birth showed that *Firmicutes* and *Proteobacteria* were the dominant phyla, in addition to *Actinomycetes* and *Bacteroides*. Interestingly, the development of resident respiratory microbiota is largely dependent on exposure in the first few hours, including mode of delivery, and the environment over the next 4–5 months. Many human clinical studies found a relation between alterations in the lung microbiota and asthma. The potential of airway microbes to regulate asthma is readily recognized due to their proximity to sites of allergic inflammation, while the GI microbiota, although anatomically isolated, are now recognized to play a vital role in asthma. The roles and dysfunction of the GI microbiota are considered to have indirect physiological effects on distal anatomical sites, including the lungs. The communication between the colonization of GI tract and the mechanism of asthma, termed the gut-lung axis, is highly complex, which stresses the interaction between the GI tract and the lung. The gut-lung axis, which in asthma mainly involves changes in the immune differentiation of cells and partial production of metabolites impacted by the mechanisms of the GI microbiota, has been confirmed. Namely, a disrupted connection within the axis or a disruption to the composition of the gut microbiota can lead to negative effects on the immunological equilibrium mechanism; in turn, this may result in allergic reactions (Durack et al., 2018; Chatenoud et al., 2020). In fact, we do not fully understand the mechanisms underlying this axis, but the GI microbiota is considered to play a crucial role in the gut-lung axis, and several contributing pathways have been confirmed (see Figure 3). Based on the existing mechanisms, we summarized the important cells in these pathways and stated their effects in Table 2 (Choy et al., 2015; Kumar et al., 2017; Cait et al., 2018).

**TABLE 2:** Important cells in these pathways and stated their effects.

Cells	Primary functions	Note	References
Treg cells	Play a crucial role immune homeostasis, particularly in allergy	Tregs modulate production of the major mucosal antibody, IgA, through the production of TGFβ	Bousquet et al., 2010
iNKT cells	Fill an important niche, bridging both innate and adaptive immune functions	The potential of gut microbiota to direct differentiation and function of immune populations in the lungs	Durack et al., 2018
Th17 cells	Maintain barrier function and clearing pathogens at mucosal surfaces	A potential therapeutic target in severe asthma	Chatenoud et al., 2020

New models for the diagnosis, phenotyping and prediction of asthma treatment responses are the result of our growing understanding of the role of symbiotic microbiota in asthma; as a result, microbial ecology can lead to new therapeutic approaches to prevent and treat asthma. Some clinical studies have shown that the main mechanism in treating allergic asthma with probiotics involves regulating the composition of the GI microbiota, binding to the receptor competitively, preventing bacterial invasion and producing bacteriocins to prevent the growth of pathogens. A meta-analysis by Lin et al. (2013) suggested that probiotics can significantly reduce episodes of asthma; nevertheless, no significant differences were obtained regarding FEV1 (i.e., forced expiratory volume in the first second), Childhood Asthma Control Test (CACT) scores, PEF (i.e., peak expiratory flow) and quality of life (Zuccotti et al., 2015). Of note, the severity of the airflow limitation, as measured by percent predicted FEV1; PEF can evaluate the degree of airway blockage and make an effective judgment on the diagnosis or recovery of asthma and the severity of the disease; and the CACT is a test questionnaire to assess asthma control in children aged 4–11 years and can be used at home for long-term monitoring of the condition. As underlined in two recent reviews, the specific effects of probiotics in children who suffer from asthma need to be further confirmed based randomized-controlled trials (RCTs) with larger sample sizes (Zuccotti et al., 2015; Huang et al., 2019). In our opinion, pharmacodynamic alteration of the GI microbiota to change its composition or to target specific host pathways impacted by the GI microbiota in asthma patients remain promising therapeutic options.

### Food Allergy

Food allergy, a severe health issue, has aroused public concern, especially in developed countries. About 5% of children in the United States who are 5 years old or younger suffer from FA, and evidence shows that SCFAs produced by the GI microbiota, dietary elements and the GI microbiota seriously affect immune tolerance (Kim et al., 2016).

This first studies to use germiculture showed that babies who were allergic to milk had a higher total bacterial and anoxybiotic microbe count. In addition, there is evidence that the dysbiosis of the GI microbiota prior to FA subsequently impacts the progression of FA. An investigation by Goldberg et al. (2020) discovered that babies with food allergies showed a lower abundance of *Prevotella copri* than babies without food allergies, with the concomitant increase in SCFAs, which may destroy intestinal homeostasis and barrier function. Currently, microbiota-directed therapy has been a new focus for the treatment of food allergies, and studies with larger sample sizes are needed to clarify the specific effects (Schuijs et al., 2015).

### Eczema

Eczema is a common chronic disease of the skin that commonly begins in infancy (Biagini Myers and Khurana Hershey, 2010). Reduced diversity of the GI microbiota has been proven by several prospective studies to be related to the morbidity of eczema. In 2012, a prospective study by Abrahamsson et al. (2012) discovered that babies who suffered from eczema at the age of one-and-a-half had a lower diversity of the GI microbiota than healthy babies. Additionally, a lower abundance of *Lactobacillus, Bifidobacterium* and SCFAs was detected in children with eczema at the age of one than in healthy babies (Song et al., 2016). Interestingly, several probiotics that mainly contain the genera *Lactobacillus* and *Bifidobacterium* can be used as therapy for eczema because they decrease IgE levels (Toh et al., 2012; Abrahamsson et al., 2014). Nevertheless, the literature on the effectiveness of probiotic use for treating eczema is limited and hampered because studies fail to address important confounding factors. In fact, more rigorous experiments and long-term follow-up are needed to prove the efficacy of this type of treatment.

### Allergic Rhinitis

Compared to other allergic diseases, there is markedly less literature specifically discussing the effect of the GI microbiota on the development of allergic rhinitis, and many of the studies that do exist are based on mouse models. A study by Flach and Diefenbach (2015). found a fixed relation between lifetime antibiotic administration and the eventual development of allergic rhinitis (OR 1.06; 95% CI 1.04–1.09) (Flach and Diefenbach, 2015). For allergic rhinitis, the GI microbiota is considered a new target for early intervention, but its mechanism and therapeutic effect need to be further studied. While there are few significant experimental results and studies correlated with allergic diseases, it is well documented that a correlation can be drawn between the GI microbiota and asthma (Bousquet et al., 2010; Suzuki et al., 2010; Shi et al., 2017; van den Elsen et al., 2017), which is frequently associated with allergic rhinitis. Thus, further research in this direction is needed.

## Conclusion and Outlook

The recent development of NGS, genomic analysis, metabolomics and proteomics has facilitated a clearer understanding of the important role of the GI microbiota in allergic diseases, and more emphasis has been placed on the significance of maintaining intestinal microbial communities. Overwhelming evidence shows that the composition of the intestinal flora of children who suffer from allergic diseases is significantly different from that of healthy children. In addition, the composition of the GI microbiota is mainly influenced by the mode of delivery, infant diet, environment, antibiotic use by the infant, etc., and the abnormal structure of the GI microbiota has been closely related to the incidence rate of allergic diseases, which provided us with a new idea that intestinal flora disorder in infancy may be considered an important predictor of allergic diseases later in life. Taken together, these findings show that an understanding of these interactions can contribute to the development of valid prevention and therapeutic strategies for allergic diseases. Probiotics can considerably reduce episodes of allergic diseases, but additional RCTs with larger sample sizes need to be conducted to evaluate the curative effect of these strategies.

## Author Contributions

TH drafted the manuscript. YD generated the figure. MZ performed the background research. MZ and QH edited the manuscript. All authors have read and approved the content of the manuscript.

## Conflict of Interest

The authors declare that the research was conducted in the absence of any commercial or financial relationships that could be construed as a potential conflict of interest.

## Publisher’s Note

All claims expressed in this article are solely those of the authors and do not necessarily represent those of their affiliated organizations, or those of the publisher, the editors and the reviewers. Any product that may be evaluated in this article, or claim that may be made by its manufacturer, is not guaranteed or endorsed by the publisher.

## Footnotes

1
https://www.worldallergy.org/

## References

[B1] Aagaard K. M. (2014). Author response to comment on “the placenta harbors a unique microbiome”. *Sci. Transl. Med.* 6:254lr3. 10.1126/scitranslmed.3010007 25232176

[B2] Abrahamsson T. R. Jakobsson H. E. Andersson A. F. Bjorksten B. Engstrand L. Jenmalm M. C. (2012). Low diversity of the gut microbiota in infants with atopic eczema. *J. Allergy Clin. Immunol.* 129 434–40, 440.e1-2. 22153774 10.1016/j.jaci.2011.10.025

[B3] Abrahamsson T. R. Jakobsson H. E. Andersson A. F. Bjorksten B. Engstrand L. Jenmalm M. C. (2014). Low gut microbiota diversity in early infancy precedes asthma at school age. *Clin. Exp. Allergy* 44 842–850. 10.1111/cea.12253 24330256

[B4] Anandan C. Nurmatov U. van Schayck O. C. Sheikh A. (2010). Is the prevalence of asthma declining? Systematic review of epidemiological studies. *Allergy* 65 152–167. 10.1111/j.1398-9995.2009.02244.x 19912154

[B5] Ardissone A. N. de la Cruz D. M. Davis-Richardson A. G. Rechcigl K. T. Li N. Drew J. C. (2014). Meconium microbiome analysis identifies bacteria correlated with premature birth. *PLoS One* 9:e90784. 10.1371/journal.pone.0090784 24614698 PMC3948723

[B6] Azad M. B. Konya T. Persaud R. R. Guttman D. S. Chari R. S. Field C. J. (2016). Impact of maternal intrapartum antibiotics, method of birth and breastfeeding on gut microbiota during the first year of life: a prospective cohort study. *BJOG* 123 983–993. 10.1111/1471-0528.13601 26412384

[B7] Bae J. M. (2018). Interpretation of the hygiene and microflora hypothesis for allergic diseases through epigenetic epidemiology. *Epidemiol. Health* 40:e2018006. 10.4178/epih.e2018006 29587338 PMC5968202

[B8] Bezirtzoglou E. Tsiotsias A. Welling G. W. (2011). Microbiota profile in feces of breast- and formula-fed newborns by using fluorescence in situ hybridization (FISH). *Anaerobe* 17 478–482. 10.1016/j.anaerobe.2011.03.009 21497661

[B9] Biagini Myers J. M. Khurana Hershey G. K. (2010). Eczema in early life: genetics, the skin barrier, and lessons learned from birth cohort studies. *J. Pediatr.* 157 704–714. 10.1016/j.jpeds.2010.07.009 20739029 PMC2957505

[B10] Bottacini F. van Sinderen D. Ventura M. (2017). Omics of bifidobacteria: research and insights into their health-promoting activities. *Biochem. J.* 474 4137–4152. 10.1042/BCJ20160756 29212851

[B11] Bousquet J. Mantzouranis E. Cruz A. A. Ait-Khaled N. Baena-Cagnani C. E. Bleecker E. R. (2010). Uniform definition of asthma severity, control, and exacerbations: document presented for the World Health Organization Consultation on Severe Asthma. *J. Allergy Clin. Immunol.* 126 926–938. 10.1016/j.jaci.2010.07.019 20926125

[B12] Brosseau C. Selle A. Duval A. Misme-Aucouturier B. Chesneau M. Brouard S. (2021). Prebiotic supplementation during pregnancy modifies the gut microbiota and increases metabolites in amniotic fluid, driving a tolerogenic environment in utero. *Front. Immunol.* 12:712614. 10.3389/fimmu.2021.712614 34335628 PMC8317504

[B13] Brugman S. Perdijk O. van Neerven R. J. Savelkoul H. F. (2015). Mucosal immune development in early life: setting the stage. *Arch. Immunol. Ther. Exp.* 63 251–268. 10.1007/s00005-015-0329-y 25666708 PMC4499104

[B14] Brumbaugh D. E. Arruda J. Robbins K. Ir D. Santorico S. A. Robertson C. E. (2016). Mode of delivery determines neonatal pharyngeal bacterial composition and early intestinal colonization. *J. Pediatr. Gastroenterol. Nutr.* 63 320–328. 10.1097/MPG.0000000000001124 27035381

[B15] Cai J. Chen Z. Wu W. Lin Q. Liang Y. (2021). High animal protein diet and gut microbiota in human health. *Crit. Rev. Food Sci. Nutr.* 10.1080/10408398.2021.1898336 Online ahead of print 33724115

[B16] Cait A. Hughes M. R. Antignano F. Cait J. Dimitriu P. A. Maas K. R. (2018). Microbiome-driven allergic lung inflammation is ameliorated by short-chain fatty acids. *Mucosal. Immunol.* 11 785–795. 10.1038/mi.2017.75 29067994

[B17] Cerdo T. Dieguez E. Campoy C. (2019). Early nutrition and gut microbiome: interrelationship between bacterial metabolism, immune system, brain structure, and neurodevelopment. *Am. J. Physiol. Endocrinol. Metab.* 317 E617–E630. 10.1152/ajpendo.00188.2019 31361544

[B18] Cereta A. D. Oliveira V. R. Costa I. P. Guimaraes L. L. Afonso J. P. R. Fonseca A. L. (2021). Early life microbial exposure and immunity training effects on asthma development and progression. *Front. Med.* 8:662262. 10.3389/fmed.2021.662262 34222279 PMC8241902

[B19] Chai G. Governale L. McMahon A. W. Trinidad J. P. Staffa J. Murphy D. (2012). Trends of outpatient prescription drug utilization in US children, 2002-2010. *Pediatrics* 130 23–31. 10.1542/peds.2011-2879 22711728

[B20] Chatenoud L. Bertuccio P. Turati F. Galeone C. Naldi L. Chatenoud L. (2020). Markers of microbial exposure lower the incidence of atopic dermatitis. *Allergy* 75 104–115. 10.1111/all.13990 31321780

[B21] Choy D. F. Hart K. M. Borthwick L. A. Shikotra A. Nagarkar D. R. Siddiqui S. (2015). TH2 and TH17 inflammatory pathways are reciprocally regulated in asthma. *Sci. Transl. Med.* 7:301ra129. 10.1126/scitranslmed.aab3142 26290411

[B22] Cox L. M. Yamanishi S. Sohn J. Alekseyenko A. V. Leung J. M. Cho I. (2014). Altering the intestinal microbiota during a critical developmental window has lasting metabolic consequences. *Cell* 158 705–721. 10.1016/j.cell.2014.05.052 25126780 PMC4134513

[B23] Crosson T. Wang J. C. Doyle B. Merrison H. Balood M. Parrin A. (2021). FcepsilonR1-expressing nociceptors trigger allergic airway inflammation. *J. Allergy Clin. Immunol.* 147 2330–2342. 10.1016/j.jaci.2020.12.644 33453289 PMC9004488

[B24] Cryan J. F. O’Riordan K. J. Cowan C. S. M. Sandhu K. V. Bastiaanssen T. F. S. Boehme M. (2019). The microbiota-gut-brain axis. *Physiol. Rev.* 99 1877–2013. 10.1152/physrev.00018.2018 31460832

[B25] Dickson R. P. Martinez F. J. Huffnagle G. B. (2014). The role of the microbiome in exacerbations of chronic lung diseases. *Lancet* 384 691–702. 10.1016/S0140-6736(14)61136-3 25152271 PMC4166502

[B26] Dominguez-Bello M. G. Costello E. K. Contreras M. Magris M. Hidalgo G. Fierer N. (2010). Delivery mode shapes the acquisition and structure of the initial microbiota across multiple body habitats in newborns. *Proc. Natl. Acad. Sci. U.S.A.* 107 11971–11975. 10.1073/pnas.1002601107 20566857 PMC2900693

[B27] Durack J. Huang Y. J. Nariya S. Christian L. S. Ansel K. M. Beigelman A. (2018). National heart, and a. blood institute’s, bacterial biogeography of adult airways in atopic asthma. *Microbiome* 6:104. 10.1186/s40168-018-0487-3 29885665 PMC5994066

[B28] Eichenfield L. F. Hanifin J. M. Beck L. A. Lemanske R. F. Jr. Sampson H. A. Weiss S. T. (2003). Atopic dermatitis and asthma: parallels in the evolution of treatment. *Pediatrics* 111 608–616. 10.1542/peds.111.3.608 12612244

[B29] Flach M. Diefenbach A. (2015). Adipose tissue: ILC2 crank up the heat. *Cell Metab.* 21 152–153. 10.1016/j.cmet.2015.01.015 25651167

[B30] Forsberg A. Abrahamsson T. R. Bjorksten B. Jenmalm M. C. (2013). Pre- and post-natal Lactobacillus reuteri supplementation decreases allergen responsiveness in infancy. *Clin. Exp. Allergy* 43 434–442. 10.1111/cea.12082 23517039

[B31] Francino M. P. (2014). Early development of the gut microbiota and immune health. *Pathogens* 3 769–790. 10.3390/pathogens3030769 25438024 PMC4243441

[B32] Francino M. P. (2018). Birth mode-related differences in gut microbiota colonization and immune system development. *Ann Nutr. Metab.* 73(Suppl. 3), 12–16. 10.1159/000490842 30041189

[B33] Gasparrini A. J. Wang B. Sun X. Kennedy E. A. Hernandez-Leyva A. Ndao I. M. (2019). Persistent metagenomic signatures of early-life hospitalization and antibiotic treatment in the infant gut microbiota and resistome. *Nat. Microbiol.* 4 2285–2297. 10.1038/s41564-019-0550-2 31501537 PMC6879825

[B34] Goldberg M. R. Mor H. Magid Neriya D. Magzal F. Muller E. Appel M. Y. (2020). Microbial signature in IgE-mediated food allergies. *Genome Med.* 12:92. 10.1186/s13073-020-00789-4 33109272 PMC7592384

[B35] Gong X. Cai Q. Liu X. An D. Zhou D. Luo R. (2021). Gut flora and metabolism are altered in epilepsy and partially restored after ketogenic diets. *Microb. Pathog.* 155:104899. 10.1016/j.micpath.2021.104899 33894293

[B36] Han X. Krempski J. W. Nadeau K. (2020). Advances and novel developments in mechanisms of allergic inflammation. *Allergy* 75 3100–3111. 10.1111/all.14632 33068299

[B37] Harmsen H. J. de Goffau M. C. (2016). The human gut microbiota. *Adv. Exp. Med. Biol.* 902 95–108. 10.1007/978-3-319-31248-4_7 27161353

[B38] Ho M. H. Lee S. L. Wong W. H. Ip P. Lau Y. L. (2012). Prevalence of self-reported food allergy in Hong Kong children and teens–a population survey. *Asian Pac. J. Allergy Immunol.* 30 275–284. 23393907

[B39] Hong S. J. Lee M. S. Sohn M. H. Shim J. Y. Han Y. S. Park K. S. (2004). Self-reported prevalence and risk factors of asthma among Korean adolescents: 5-year follow-up study, 1995-2000. *Clin. Exp. Allergy* 34 1556–1562. 10.1111/j.1365-2222.2004.02084.x 15479270

[B40] Huang L. Guo J. Li W. Jiang M. Wang F. Kang J. (2019). Probiotics, prebiotics, and synbiotics for the treatment of asthma: protocol for a systematic review. *Medicine* 98:e17840. 10.1097/MD.0000000000017840 31764780 PMC6882644

[B41] Jakobsson H. E. Abrahamsson T. R. Jenmalm M. C. Harris K. Quince C. Jernberg C. (2014). Decreased gut microbiota diversity, delayed Bacteroidetes colonisation and reduced Th1 responses in infants delivered by caesarean section. *Gut* 63 559–566. 10.1136/gutjnl-2012-303249 23926244

[B42] Jimenez E. Marin M. L. Martin R. Odriozola J. M. Olivares M. Xaus J. (2008). Is meconium from healthy newborns actually sterile? *Res. Microbiol.* 159 187–193. 10.1016/j.resmic.2007.12.007 18281199

[B43] Justiz Vaillant A. A. Vashisht R. Zito P. M. (2021). *Immediate Hypersensitivity Reactions.* Treasure Island, FL: StatPearls Publishing. 30020687

[B44] Kim K. S. Hong S. W. Han D. Yi J. Jung J. Yang B. G. (2016). Dietary antigens limit mucosal immunity by inducing regulatory T cells in the small intestine. *Science* 351 858–863. 10.1126/science.aac5560 26822607

[B45] Korpela K. Salonen A. Virta L. J. Kekkonen R. A. Forslund K. Bork P. (2016). Intestinal microbiome is related to lifetime antibiotic use in Finnish pre-school children. *Nat. Commun.* 7:10410. 10.1038/ncomms10410 26811868 PMC4737757

[B46] Kumar A. Suryadevara N. Hill T. M. Bezbradica J. S. Van Kaer L. Joyce S. (2017). Natural killer T cells: an ecological evolutionary developmental biology perspective. *Front. Immunol.* 8:1858. 10.3389/fimmu.2017.01858 29312339 PMC5743650

[B47] Laubereau B. Filipiak-Pittroff B. von Berg A. Grubl A. Reinhardt D. Wichmann H. E. (2004). Caesarean section and gastrointestinal symptoms, atopic dermatitis, and sensitisation during the first year of life. *Arch Dis Child* 89 993–997. 10.1136/adc.2003.043265 15499049 PMC1719727

[B48] Lee E. Kim B. J. Kang M. J. Choi K. Y. Cho H. J. Kim Y. (2016). Dynamics of gut microbiota according to the delivery mode in healthy korean infants. *Allergy Asthma Immunol. Res.* 8 471–477. 10.4168/aair.2016.8.5.471 27334787 PMC4921703

[B49] Lee Y. K. Mazmanian S. K. (2010). Has the microbiota played a critical role in the evolution of the adaptive immune system? *Science* 330 1768–1773. 10.1126/science.1195568 21205662 PMC3159383

[B50] Lin A. Bik E. M. Costello E. K. Dethlefsen L. Haque R. Relman D. A. (2013). Distinct distal gut microbiome diversity and composition in healthy children from Bangladesh and the United States. *PLoS One* 8:e53838. 10.1371/journal.pone.0053838 23349750 PMC3551965

[B51] Maynard C. L. Elson C. O. Hatton R. D. Weaver C. T. (2012). Reciprocal interactions of the intestinal microbiota and immune system. *Nature* 489 231–241. 10.1038/nature11551 22972296 PMC4492337

[B52] McArdle A. J. Vito O. Patel H. Seaby E. G. Shah P. Wilson C. (2021). Treatment of multisystem inflammatory syndrome in children. *N. Engl. J. Med* 385 11–22. 10.1056/NEJMoa2102968 34133854 PMC8220965

[B53] McKenzie C. Tan J. Macia L. Mackay C. R. (2017). The nutrition-gut microbiome-physiology axis and allergic diseases. *Immunol. Rev.* 278 277–295. 10.1111/imr.12556 28658542

[B54] Milani C. Duranti S. Bottacini F. Casey E. Turroni F. Mahony J. (2017). the first microbial colonizers of the human gut: composition, activities, and health implications of the infant gut microbiota. *Microbiol. Mol. Biol. Rev.* 81:e36-17. 10.1128/MMBR.00036-17 29118049 PMC5706746

[B55] Mshvildadze M. Neu J. Shuster J. Theriaque D. Li N. Mai V. (2010). Intestinal microbial ecology in premature infants assessed with non-culture-based techniques. *J. Pediatr.* 156 20–25. 10.1016/j.jpeds.2009.06.063 19783002 PMC3628625

[B56] Munyaka P. M. Khafipour E. Ghia J. E. (2014). External influence of early childhood establishment of gut microbiota and subsequent health implications. *Front. Pediatr.* 2:109. 10.3389/fped.2014.00109 25346925 PMC4190989

[B57] Nanthakumar N. N. Fusunyan R. D. Sanderson I. Walker W. A. (2000). Inflammation in the developing human intestine: a possible pathophysiologic contribution to necrotizing enterocolitis. *Proc. Natl. Acad. Sci. U.S.A.* 97 6043–6048. 10.1073/pnas.97.11.6043 10823949 PMC18555

[B58] Noverr M. C. Huffnagle G. B. (2005). The ‘microflora hypothesis’ of allergic diseases. *Clin. Exp. Allergy* 35 1511–1520. 10.1111/j.1365-2222.2005.02379.x 16393316

[B59] Nwaru B. I. Virtanen S. M. (2017). Allergenic food introduction and childhood risk of allergic or autoimmune disease. *JAMA* 317:86. 10.1001/jama.2016.18329 28030695

[B60] Praveen P. Jordan F. Priami C. Morine M. J. (2015). The role of breast-feeding in infant immune system: a systems perspective on the intestinal microbiome. *Microbiome* 3:41. 10.1186/s40168-015-0104-7 26399409 PMC4581423

[B61] Prince A. L. Ma J. Kannan P. S. Alvarez M. Gisslen T. Harris R. A. (2016). The placental membrane microbiome is altered among subjects with spontaneous preterm birth with and without chorioamnionitis. *Am. J. Obstet. Gynecol.* 214 627.e1–627.e16. 10.1016/j.ajog.2016.01.193 26965447 PMC4909356

[B62] Rodriguez J. M. Murphy K. Stanton C. Ross R. P. Kober O. I. Juge N. (2015). The composition of the gut microbiota throughout life, with an emphasis on early life. *Microb Ecol. Health Dis.* 26:26050. 10.3402/mehd.v26.26050 25651996 PMC4315782

[B63] Romagnani S. (2006). Regulation of the T cell response. *Clin. Exp. Allergy* 36 1357–1366. 10.1111/j.1365-2222.2006.02606.x 17083345

[B64] Rook G. A. Adams V. Hunt J. Palmer R. Martinelli R. Brunet L. R. (2004). Mycobacteria and other environmental organisms as immunomodulators for immunoregulatory disorders. *Springer Semin. Immunopathol.* 25 237–255. 10.1007/s00281-003-0148-9 15007629

[B65] Round J. L. Mazmanian S. K. (2010). Inducible Foxp3+ regulatory T-cell development by a commensal bacterium of the intestinal microbiota. *Proc. Natl. Acad. Sci. U.S.A.* 107 12204–12209. 10.1073/pnas.0909122107 20566854 PMC2901479

[B66] Satokari R. Gronroos T. Laitinen K. Salminen S. Isolauri E. (2009). Bifidobacterium and lactobacillus DNA in the human placenta. *Lett. Appl. Microbiol* 48 8–12. 10.1111/j.1472-765X.2008.02475.x 19018955

[B67] Schuijs M. J. Willart M. A. Vergote K. Gras D. Deswarte K. Ege M. J. (2015). Farm dust and endotoxin protect against allergy through A20 induction in lung epithelial cells. *Science* 349 1106–1110. 10.1126/science.aac6623 26339029

[B68] Segal L. N. Alekseyenko A. V. Clemente J. C. Kulkarni R. Wu B. Gao Z. (2013). Enrichment of lung microbiome with supraglottic taxa is associated with increased pulmonary inflammation. *Microbiome* 1:19. 10.1186/2049-2618-1-19 24450871 PMC3971609

[B69] Shamji M. H. Valenta R. Jardetzky T. Verhasselt V. Durham S. R. Wurtzen P. A. (2021). The role of allergen-specific IgE, IgG and IgA in allergic disease. *Allergy* 10.1111/all.14908 Online ahead of print 33999439 PMC8601105

[B70] Shi N. Li N. Duan X. Niu H. (2017). Interaction between the gut microbiome and mucosal immune system. *Mil. Med. Res.* 4:14. 10.1186/s40779-017-0122-9 28465831 PMC5408367

[B71] Song H. Yoo Y. Hwang J. Na Y. C. Kim H. S. (2016). Faecalibacterium prausnitzii subspecies-level dysbiosis in the human gut microbiome underlying atopic dermatitis. *J. Allergy Clin. Immunol.* 137 852–860. 10.1016/j.jaci.2015.08.021 26431583

[B72] Stecher B. Maier L. Hardt W. D. (2013). ‘Blooming’ in the gut: how dysbiosis might contribute to pathogen evolution. *Nat Rev. Microbiol.* 11 277–284. 10.1038/nrmicro2989 23474681

[B73] Stiemsma L. T. Reynolds L. A. Turvey S. E. Finlay B. B. (2015). The hygiene hypothesis: current perspectives and future therapies. *Immunotargets Ther.* 4 143–157. 10.2147/ITT.S61528 27471720 PMC4918254

[B74] Strachan D. P. (1989). Hay fever, hygiene, and household size. *BMJ* 299 1259–1260. 10.1136/bmj.299.6710.1259 2513902 PMC1838109

[B75] Suzuki K. Kawamoto S. Maruya M. Fagarasan S. (2010). GALT: organization and dynamics leading to IgA synthesis. *Adv. Immunol.* 107 153–185. 10.1016/B978-0-12-381300-8.00006-X 21034974

[B76] Teo S. M. Mok D. Pham K. Kusel M. Serralha M. Troy N. (2015). The infant nasopharyngeal microbiome impacts severity of lower respiratory infection and risk of asthma development. *Cell Host Microbe.* 17 704–715. 10.1016/j.chom.2015.03.008 25865368 PMC4433433

[B77] Theis K. R. Romero R. Winters A. D. Greenberg J. M. Gomez-Lopez N. Alhousseini A. (2019). Does the human placenta delivered at term have a microbiota? Results of cultivation, quantitative real-time PCR, 16S rRNA gene sequencing, and metagenomics. *Am. J. Obstet. Gynecol.* 220 .e1–.e267. 10.1016/j.ajog.2018.10.018 30832984 PMC6733039

[B78] Tierney B. T. Yang Z. Luber J. M. Beaudin M. Wibowo M. C. Baek C. (2019). The landscape of genetic content in the gut and oral human microbiome. *Cell Host Microbe* 26 283.e8–295.e8. 10.1016/j.chom.2019.07.008 31415755 PMC6716383

[B79] Toh Z. Q. Anzela A. Tang M. L. Licciardi P. V. (2012). Probiotic therapy as a novel approach for allergic disease. *Front. Pharmacol.* 3:171. 10.3389/fphar.2012.00171 23049509 PMC3448073

[B80] van den Elsen L. W. Poyntz H. C. Weyrich L. S. Young W. Forbes-Blom E. E. (2017). Embracing the gut microbiota: the new frontier for inflammatory and infectious diseases. *Clin Transl Immunology* 6:e125. 10.1038/cti.2016.91 28197336 PMC5292562

[B81] Vangay P. Ward T. Gerber J. S. Knights D. (2015). Antibiotics, pediatric dysbiosis, and disease. *Cell Host. Microbe* 17 553–564. 10.1016/j.chom.2015.04.006 25974298 PMC5555213

[B82] Wang X. Y. Zhuang Y. Ma T. T. Zhang B. Wang X. Y. (2018). Prevalence of self-reported food allergy in six regions of inner mongolia, northern china: a population-based survey. *Med. Sci. Monit.* 24 1902–1911. 10.12659/MSM.908365 29605827 PMC5894567

[B83] Wong G. W. Leung T. F. Ko F. W. (2013). Changing prevalence of allergic diseases in the Asia-pacific region. *Allergy Asthma Immunol. Res.* 5 251–257. 10.4168/aair.2013.5.5.251 24003381 PMC3756171

[B84] Wright E. K. Kamm M. A. Teo S. M. Inouye M. Wagner J. Kirkwood C. D. (2015). Recent advances in characterizing the gastrointestinal microbiome in Crohn’s disease: a systematic review. *Inflamm. Bowel. Dis.* 21 1219–1228. 10.1097/MIB.0000000000000382 25844959 PMC4450900

[B85] Zhao J. Bai J. Shen K. L. Xiang L. Huang Y. Huang S. (2011). [Questionnaire-based survey of allergic diseases among children aged 0 - 14 years in the downtown of Beijing, Chongqing and Guangzhou]. *Zhonghua Er Ke Za Zhi* 49 740–744. 22321178

[B86] Zuccotti G. Meneghin F. Aceti A. Barone G. Callegari M. L. Di Mauro A. (2015). Probiotics for prevention of atopic diseases in infants: systematic review and meta-analysis. *Allergy* 70 1356–1371. 10.1111/all.12700 26198702

